# Recognition of Abnormal-Laying Hens Based on Fast Continuous Wavelet and Deep Learning Using Hyperspectral Images

**DOI:** 10.3390/s23073645

**Published:** 2023-03-31

**Authors:** Xing Qin, Chenxiao Lai, Zejun Pan, Mingzhong Pan, Yun Xiang, Yikun Wang

**Affiliations:** 1 Zhejiang Key Laboratory of Large-Scale Integrated Circuit Design, Hangzhou Dianzi University, Hangzhou 310018, China; 2 Key Laboratory of Gravitational Wave Precision Measurement of Zhejiang Province, School of Physics and Photoelectric Engineering, Hangzhou Institute for Advanced Study, University of Chinese Academy of Sciences, Hangzhou 310024, China; 3 Agriculture Science Research Institute, Jinhua 321000, China

**Keywords:** laying hens, hyperspectral, fast continuous wavelet, deep learning, vertex component analysis

## Abstract

The egg production of laying hens is crucial to breeding enterprises in the laying hen breeding industry. However, there is currently no systematic or accurate method to identify low-egg-production-laying hens in commercial farms, and the majority of these hens are identified by breeders based on their experience. In order to address this issue, we propose a method that is widely applicable and highly precise. First, breeders themselves separate low-egg-production-laying hens and normal-laying hens. Then, under a halogen lamp, hyperspectral images of the two different types of hens are captured via hyperspectral imaging equipment. The vertex component analysis (VCA) algorithm is used to extract the cockscomb end member spectrum to obtain the cockscomb spectral feature curves of low-egg-production-laying hens and normal ones. Next, fast continuous wavelet transform (FCWT) is employed to analyze the data of the feature curves in order to obtain the two-dimensional spectral feature image dataset. Finally, referring to the two-dimensional spectral image dataset of the low-egg-production-laying hens and normal ones, we developed a deep learning model based on a convolutional neural network (CNN). When we tested the model’s accuracy by using the prepared dataset, we found that it was 0.975 percent accurate. This outcome demonstrates our identification method, which combines hyperspectral imaging technology, an FCWT data analysis method, and a CNN deep learning model, and is highly effective and precise in laying-hen breeding plants. Furthermore, the attempt to use FCWT for the analysis and processing of hyperspectral data will have a significant impact on the research and application of hyperspectral technology in other fields due to its high efficiency and resolution characteristics for data signal analysis and processing.

## 1. Introduction

The poultry industry is currently moving toward digital, intelligent, and large-scale farms that have become increasingly automated [1]. However, farmers are facing challenges related to identifying low-laying hens, analyzing problems in a timely manner, reducing feed consumption, and improving breeding efficiency. Currently, these issues are mainly resolved manually, which is inefficient and unable to satisfy the requirements of large-scale farms. Hence, enterprises that breed laying hens urgently need intelligent technology to identify and dispose of laying hens with low-egg production. Unfortunately, there are few studies on the intelligent identification of laying hens with low-egg production, and the relevant research primarily focuses on the identification of sick and dead chickens [2].

Most of the research on the automatic identification of sick and dead chickens is based on visible light imaging, and machine vision algorithms are frequently used in image processing [3]. Given that the differences in the external characteristics between low- and high-laying hens are not as great as the differences among sick, dead, and healthy hens, and the fact that visible light imaging is susceptible to problems, such as changes in ambient light conditions in the farm environment, color contrasting between the background and foreground, and shading [4,5,6], it is difficult to distinguish between low-laying hens from high-laying hens by using visible light-based imaging. On the other hand, it is not practical since various processes based on machine learning systems, such as segmentation, feature extraction, and selection, are also time-consuming and subjectively difficult tasks [7]. In recent years, computer vision systems based on deep learning have been used more and more frequently to identify individual sick and dead chickens [8]. Because they are able to process images directly, they do a smaller amount of work and are often more accurate than machine learning [4]. A two-stage deep learning-based method of identifying sick chickens was proposed by Chen et al. [9]. First, it locates the chicken’s head and body regions by using the modified Faster RCNN model. Then, it extracts the deep features from the aforementioned three image blocks and a cascade of regional feature vectors. The fusion feature classification is most effective in the comparison experiment of identifying sick chickens, with an accuracy of 88.41% [10]. A sick chicken automatic detection system based on the ResNet residual network was proposed by Zhang and Chen et al. [10]. An improved ResNet-FPN sick chicken recognition model was designed to adapt to various recognition environments after improving the network structure of ResNet. In addition to image recognition, sensors combined with machine learning algorithms have also been used to identify dead and sick chickens. For example, Bao and Lu proposed an artificial intelligence-based sensor detection method [11]. The three-dimensional total variance is intended to represent the intensity of the chicken’s activity. The maximum displacement of the chicken’s activity is measured through the foot ring fixed on each chicken. The detection terminal gathers the foot ring’s sensing data via the ZigBee network before using machine learning algorithms to determine the chicken’s condition (dead or sick). This method, however, may not be extended to distinguish laying hens with low-egg production from ones with high-egg production because the differences between low- and high-laying hens are not as obvious as those between dead and healthy chickens. There are also direct laying hen monitoring methods, mainly facilitated through the data analysis of the number of eggs produced by the chicken to infer the chicken’s condition. For instance, in a prior study, researchers used egg production data from 24 flocks of 478,919 laying hens to identify and forewarn early issues in the commercial egg production curve [12]. The individual chicken in the cage cannot be precisely identified if using this analysis method; it can only be used to identify the condition of the entire group of chickens in the cage.

In conclusion, there is no relevant, intelligent identification technology to identify laying hens with low-egg production. It is also difficult to apply the relevant technologies that are currently applied to intelligently distinguish between sick and dead laying hens or between laying hens with low-egg production and high-egg production. The external characteristics and behaviors [13] of laying hens with high- and low-egg production are distinct due to differences in the reproductive functions of laying hens during the laying period. High-egg-production laying hens typically have bright red, elastic combs and beards, while low-egg-production laying hens have lusterless, relatively pale combs. High-egg-production laying hens also tend to have combs that are relatively large and primarily inclined to one side, while those of low-laying hens are dried, shrunken, and smaller. In the review mentioned above, some researchers have adopted image recognition and machine vision algorithms to classify healthy chickens, dead chickens, and sick chickens based on the color and shape differences of chicken combs in various health states. We assume that, since the combs of these two laying hens differ visibly, the hyperspectral imaging of their combs should also differ in accordance with the fundamentals and features of hyperspectral imaging. This distinction can then, in return, be used to easily distinguish between laying hens with high- and low-egg production. Based on the above assumptions, this study is devoted to the development of a model to achieve the high-precision recognition of laying hens with normal- and low-egg production by analyzing the spectral data collected by hyperspectral sensors. At the same time, we try to present a new general method for analyzing and processing hyperspectral data, which provides important research value for the application of hyperspectral technology in other fields.

## 2. Materials and Methods

**Figure 1:**
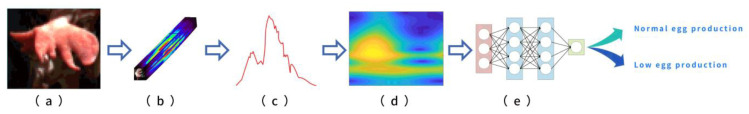
Flow chart of the experiment: (**a**) hyperspectral image; (**b**) spectral cube; (**c**) spectral curve of combs; (**d**) wavelet transform feature map; (**e**) convolutional neural network. The spectral data were obtained from the hyperspectral image to establish the spectral cube, and the one-dimensional spectral characteristic curve of the comb was extracted. The two-dimensional feature map was generated by FCWT and input into the convolutional neural network to obtain a two-classification model that can recognize normal- and abnormal-laying hens.

This study proposes a model that combines hyperspectral detection, fast continuous wavelet transform (FCWT), and a convolutional neural network (CNN) for hyperspectral feature analysis. The end member spectrum of the combs of two different types of hens was extracted by using the VCA algorithm after we first acquired the hyperspectral image data of the comb. In order to design a deep learning model that can determine whether or not hens are producing eggs normally through the comb spectrum, the one-dimensional end member hyperspectral curve was converted by FCWT into a two-dimensional hyperspectral feature matrix with more obvious features and higher resolution [14], and then it was transformed into the convolutional neural network [15,16]. We give the experimental flow chart in Figure 1.

### 2.1. Hyperspectral Data Acquisition

A traditional color camera can only record red, green, and blue images in three channels, and each channel has a very large bandwidth. When compared with traditional color cameras, hyperspectral imaging uses both imaging and hyperspectral technology. Hyperspectral technology, which can typically produce 100–400 spectral channels, is characterized by high resolution, a large number of wavebands, and narrow wavelengths. Hyperspectral imaging sensors can take multiple neighboring images with narrow wavelengths within a specific spectrum, each with more subtle details. Hyperspectral imaging analyzes the spatial and spectral features of objects by taking pictures at various wavelengths. Therefore, more subtle differences between objects can be found by using hyperspectral imaging technology [17]. The spectrum of sunlight is also referred to as the full spectrum because it contains ultraviolet, visible, and infrared spectra. The halogen lamp’s spectral distribution closely resembles the sunlight that falls on the surface of the Earth. In this experiment, we made the decision to collect the comb spectrum while it was illuminated by a halogen lamp in order to obtain the complete comb spectrum. We used the HY-6010 handheld hyperspectral imager from HHIT, which ranges from 400 to 1000 nm with 300 spectral channels.

In a layer farm in Lanxi, Zhejiang Province, China, breeders manually selected 300 hens with normal-, low-, or no-egg production. Among them, the ratio of normal-egg-laying chickens to low- or no-egg-laying chickens was 7:3. The hyperspectral imaging sensors produced hyperspectral image data. The values measured by the hyperspectral imaging sensor were stored in binary data files by using band sequential (BSQ), band per pixel interleaved (BIP), or band per line interleaved (BIL) encoding formats. In this experiment, the measured values of the hyperspectral data were sequentially stored in BIL coding format. The values read from the data file were arranged in a three-dimensional array in the form of X Y L for the processing of hyperspectral image data, where X and Y were the spatial dimensions of the collected data, and L was the spectral dimension, which specified the number of spectral bands used in the acquisition process. The three-dimensional array could therefore be thought of as a collection of two-dimensional monochromatic images taken at various wavelengths. This set of data was called the hyperspectral data cube or data cube. In our experiments, the number of bands acquired was L = 300. Our preprocessing of hyperspectral images includes data cropping, data filtering, and reflectance correction. The data filtering improves the accuracy of the extracted information, and the reflectivity correction eliminates the influence of shadow and light on the reflectivity to obtain the actual reflectivity information of the comb. We use ENVI I^®^ to convert the chicken comb hyperspectral image (Figure 1a) into a spectral cube with more obvious spectral information (Figure 1b).

In the experiment, we used the spectral curves of 400 chicken combs as the training dataset for the CNN. We then fed the dataset and labels into the CNN for learning. Additionally, we divided the set of chicken comb spectral data in a 4:1 ratio. The specific distribution of the dataset is provided in Table 1.

### 2.2. Data Processing

#### 2.2.1. Automatic and Fast Extraction of Hyperspectral End Member of Comb Based on VCA

The pixels in hyperspectral data are generally mixed pixels that contain multiple materials, while an end member only has information about one material. To describe the various end members that are present in each pixel, the mixed pixels can be decomposed, and several end members that are mixed into each pixel can be identified.

VCA algorithm is proposed by Nascimento et al. [18]. Unsupervised linear decomposition or linear spectral mixture analysis was carried out by the VCA algorithm on a set of mixed hyperspectral vector data. The end member spectral features, also known as pure spectral features that are present in hyperspectral data, can be extracted using this technique.

In this paper, we used the VCA algorithm to extract the spectral features of the chicken crown end member because of its low computational complexity, quick speed, and high accuracy. In the following sections, we will provide a brief overview of the algorithm’s application.

In the case of linear mixing of the given hyperspectral data, the hyperspectral vector is given by Formula (1):

(1) $$r=\left[r_{1},r_{2},\ldots ,r_{L}\right]=x+n=M\gamma \alpha +n,$$

where $r=\left[r_{1},r_{2},\ldots ,r_{L}\right]$ is a vector, and its dimension L is the number of bands of the spectrum. $M=\left[M_{1},M_{2},\ldots ,M_{p}\right]$ denotes a mixed matrix containing p end members, where M_i_ is the characteristic of the ith end member. γ is the scaling factor caused by changes in surface illumination. $\alpha ={\left[\alpha_{1},\alpha_{2},\ldots ,\alpha_{p}\right]}^{T}$ represents the abundance vector corresponding to each end member, and n represents the additional noise of the system.

Assuming there is no noise, r represents the vector in the dimension p subspace Ep of the convex cone Cp. The VCA algorithm first uses singular value decomposition (SVD) to identify the subspace Ep and then uses the formula:

(2) $$y=r/\left(r^{T}u\right)$$

to project the points in the convex cone Cp onto the simplex SP. The affine transformation set with dimension p − 1 contains the simplex SP. Additionally, the simplex with the same vertex, resulting from the projection of the convex cone Cp in the subspace Ed of any dimension contained in Ep, serves as the end member that needs to be extracted.

**Figure 2:**
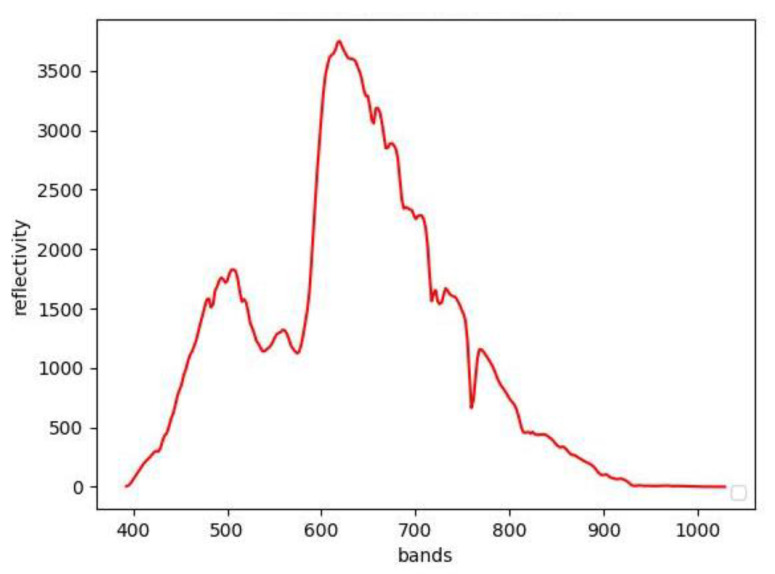
End member spectral curve of hens’ comb: The *x*-axis is the wavelength of light, and the *y*-axis is the reflectivity of light at the corresponding wavelength. The spectral curve describes the spectral characteristics of the comb.

**Figure 3:**
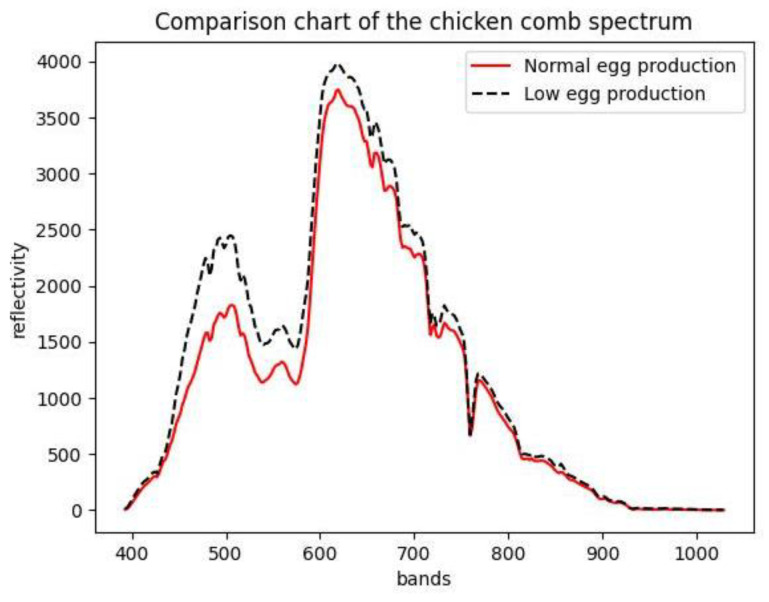
Comparison of end member spectral curves of normal- and low-egg production hens’ comb: Although the overall shape of the hens’ comb spectral curve tends to be consistent, there are still small differences between the curves of normal hens and abnormal hens. The slope of the spectral reflectance of abnormal chickens in the 450–600 band is greater than that of normal chickens.

We can quickly extract the chicken comb end member spectra of two kinds of chickens from hyperspectral data using the VCA algorithm. The end member spectral curve of hen’s comb is shown in Figure 2. We compared the two cockscomb end member spectra, plotting them into curves (Figure 3). As can be seen in the 400–600 band, the reflectance of the comb end member spectral curve with low egg production is higher than that of the cockscomb end member spectral curve with normal egg production.

#### 2.2.2. The Fast Continuous Wavelet Transformation

Although the Fourier transform is a powerful tool for data analysis, it has certain limitations. One of these limitations is that, since the data are represented as the sum of sine waves, it cannot accurately represent mutations. The sine wave is not stationary in time or space, and it constantly oscillates. Therefore, a new function with good localization properties in the time-frequency domain is required. This is where the wavelet comes in, which is a wave-like oscillation with zero mean value and rapid attenuation. Unlike sine waves, wavelets only last for a finite amount of time and come in various sizes and shapes. This makes wavelet analysis an excellent tool for a broad range of applications. By using the wavelet transform, a signal can be analyzed at different scales, allowing for a more precise analysis of the data. The wavelet transform can be used to perform a multi-scale localization analysis of the signal, and the signal can be gradually refined through scaling and translation operations. This approach can focus on any signal details, solve the challenging Fourier transform problem, and fully emphasize the signal’s features. In this paper, the wavelet transform is primarily used to refine the features of spectral signals to improve the resolution and differences of the two comb spectral signal analysis results.

In wavelet analysis, the mother wavelet expression is obtained by performing a Fourier transform on the determined Morlet wavelet expression [19]:

(3) $$\psi \left(t\right)=e^{\left({i\omega }_{0}t\right)}e^{\left(-\frac{t^{2}}{2}\right)}$$

(4) $$\hat{\psi \left(\omega \right)}=\sqrt{2\pi }e^{\left(-\frac{{\left(\omega -\omega_{0}\right)}^{2}}{2}\right)}$$

According to the mother wavelet expression, we can obtain its frequency window width:

(5) $$△\omega_{\hat{\psi \left(\omega \right)}}=\frac{1}{{\left[\int_{-\infty }^{+\infty }{\left|\hat{\psi \left(\omega \right)}\right|}^{2}d\omega \right]}^{1/2}}{\left[\int_{-\infty }^{+\infty }{\left(\omega -\omega_{0}\right)}^{2}{\left|\hat{\psi \left(\omega \right)}\right|}^{2}d\omega \right]}^{1/2}$$

Through the wavelet expression, we carry out Fourier transform to obtain the expression of the wavelet in the frequency domain:

(6) $$\psi_{j,k}\left(t\right)=2^{\frac{-j}{2}}\psi \left(2^{j}t-k\right)$$

(7) $${\hat{\psi }}_{\alpha_{\left(k\right)}}\left(\omega \right)=\hat{\psi }\left(\alpha_{\left(k\right)}\omega \right)$$

After the Fourier transform of the hyperspectral signal:

(8) $$\hat{X}\left(\xi \right)=\mathrm{FFT}\left[X\left(k\right)\right]$$

Generate a sequence $\alpha_{\left(k\right)}$ of n scales in the $\left[2^{a},2^{b}\right]$ range. The subwavelet center frequency corresponding to each scale is $\omega_{\alpha_{\left(k\right)}}=\frac{1}{\alpha_{\left(k\right)}}\omega_{0}$. The nonzero frequency window is $△\omega_{\alpha_{\left(k\right)}}=\frac{1}{\alpha_{\left(k\right)}}△\omega_{\hat{\psi \left(\omega \right)}}$. In the nonzero frequency window $△\omega_{\alpha_{\left(k\right)}}$, The intermediate value H obtained by multiplying $\hat{X}\left(\xi \right)$ with ${\hat{\psi }}_{\alpha_{\left(k\right)}}\left(\omega \right)$ signal after subwavelet Fourier transform is transformed into wavelet coefficient W by inverse Fourier transform.

(9) $$H={\hat{\psi }}_{\alpha_{\left(k\right)}}\left(\omega \right)\;\cdot \;\hat{X}\left(\xi \right)$$

(10) $$W=\mathrm{IFFT}\left[{\hat{\psi }}_{\alpha_{\left(k\right)}}\left(\omega \right)\;\cdot \;\hat{X}\left(\xi \right)\right]$$

**Figure 4:**
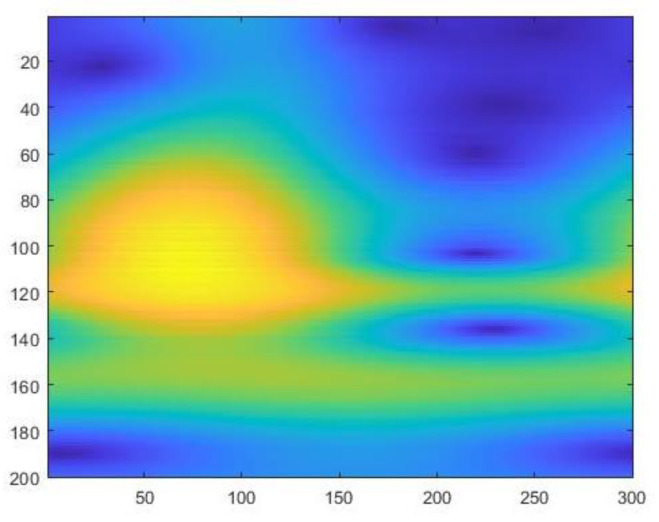
Two-dimensional hyperspectral feature map of low egg production; It can be observed that the area of the yellow part is small, and the color is light green in the range of ordinate 140–180.

**Figure 5:**
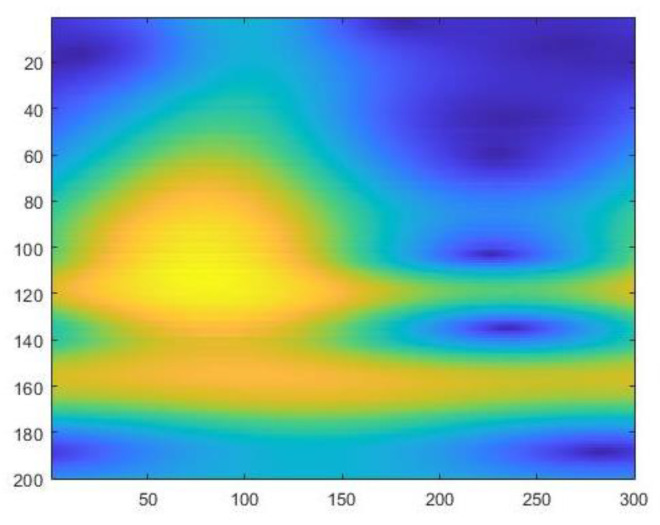
Two-dimensional hyperspectral feature map of normal-egg production: It can be observed that the yellow part has a large area, and the color is orange in the range of the ordinate 140–180.

The feature matrix is obtained after using the wavelet transform by sequentially stacking the wavelet coefficient W obtained by each scale on n scales. FCWT converts the spectral curve from a one-dimensional version to a two-dimensional hyperspectral feature map. A 200 × 300 matrix makes up each two-dimensional hyperspectral feature map. It is a two-dimensional spectral feature map of combs from chickens with low-egg production and a two-dimensional spectral feature map of combs from chickens with normal-egg production, as shown in Figure 4 and Figure 5 below.

#### 2.2.3. Convolutional Neural Network

A type of feedforward neural network with a deep structure and convolution calculation is known as a convolutional neural network (CNN). It can perform representational learning and possesses the qualities of local connection and weight sharing. The convolutional neural network is one of the most widely used models and one of the representative deep learning algorithms.

The hyperspectral feature map of chicken comb after a wavelet transform is used in this study to tackle the binary classification problem of normal and low egg production by using convolutional neural networks. An input layer, a convolution layer, an activation function, a pooling layer, a fully connected layer, an output layer, etc., are typically present in convolutional neural networks. The most crucial layer in a convolutional neural network is the convolutional layer, which is also where the convolutional neural network gets its name. Convolution operations are used to extract various features from input data. The convolution layer’s job is to convolve the input data, which is another way of saying that it performs the filtering. A window filter is a convolution kernel. A custom-sized convolution kernel is used as a sliding window to convolve the input data during the network training process. The most widely used activation functions at the moment are Relu, Tanh, Sigmoid, etc. The Relu function, which has a quick convergence and a straightforward gradient, was used in this study. The pooling layer’s primary duties are to facilitate optimization and avoid over-fitting. Usually, a feature with a lot of dimensions is obtained after the convolution layer. To create a new feature with a smaller dimension, the feature is divided into regions, and the maximum value is taken from each region. The pooling procedure also involves a sliding window that moves across the input matrix. The maximum value of the data matrix in the window is used as the output in the sliding process. The pooling layer is 2 × 2 in size, and each step is 1 in height. The fully connected layer’s function is to combine all local features, extract their correlation through nonlinear changes, and then transform them into global features. The fully connected layer is typically used as the convolutional neural network’s top layer.

**Figure 6:**
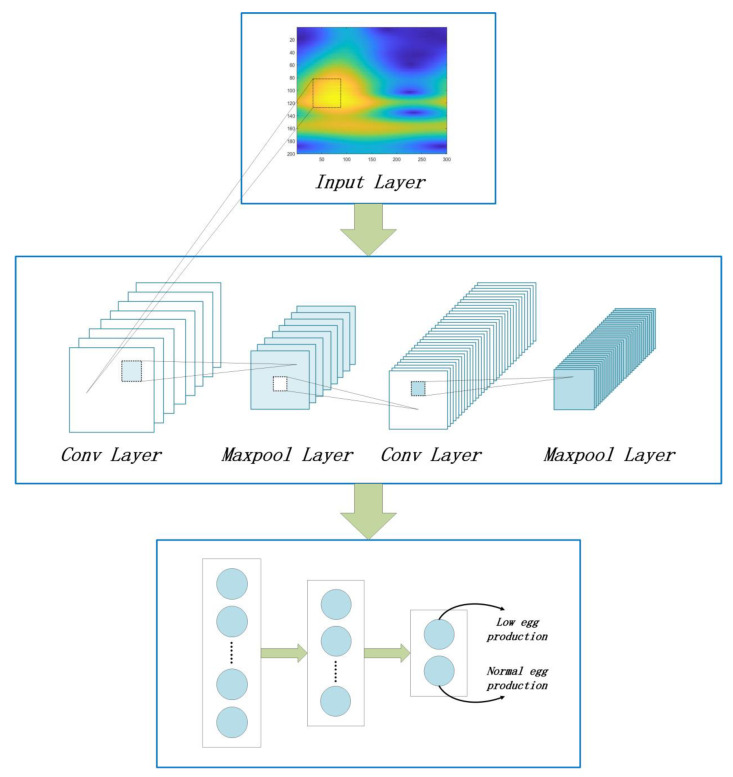
Convolutional neural network composition diagram: The two-dimensional hyperspectral feature map is put into the input layer. After two convolution and pooling layers, the feature is extracted and input into the fully connected layer, and finally obtains a binary classification model.

We converted the collected end member spectral dataset into a new dataset of two-dimensional spectral feature images using wavelet transformation. The training set and verification set were then fed into the convolutional neural network for training, divided into a 4:1 ratio. The convolutional neural network in this study contained two convolutional layers and a fully connected layer that contained the parameters. The data is flattened by the Flatten layer and then fed into the fully connected layer after two layers of convolution and pooling. The convolutional neural network’s composition diagram is shown in Figure 6, and the specifics of parameter selection for each layer are provided in Table 2.

## 3. Results

**Figure 7:**
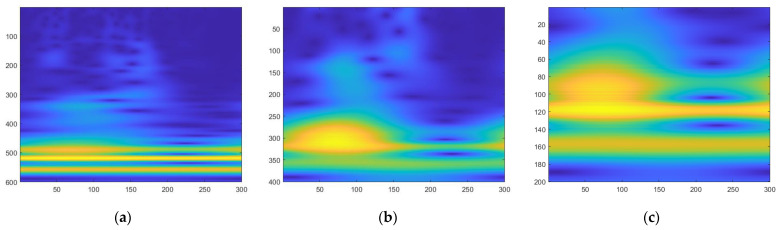
Wavelet transform images under different scales and $\omega_{0}$ have different graphic results; choosing the appropriate scale is helpful to the mining of signal details: (**a**) image at scale $\left[2,2^{6}\right]$, $\omega_{0}\;$= 12; (**b**) image at scale $\left[2^{3},2^{6}\right]$, $\omega_{0}\;$= 8; (**c**) image at scale $\left[2^{5},2^{6}\right]$, $\omega_{0}\;$= 10.

The ability of a wavelet to mine signal details is different in different scale ranges and different Morlet wavelet $\omega_{0}$. When performing FCWT, we usually choose Morlet wavelets with different $\omega_{0}$ for different signals. When the signal changes gently or the frequency is low, we should choose a smaller $\omega_{0}$. When the signal is steep or the oscillation frequency is high, $\omega_{0}$ should be appropriately increased. In different scale ranges, the feature maps generated by the FCWT results also correspond to the characteristics in different frequency ranges. When a small-scale range is selected, the characteristics of the feature map in a specific scale range that corresponds to the frequency range can be amplified. When a large-scale range is selected, a more comprehensive feature map of the frequency range is obtained. Figure 7 is the result of the same spectral curve generated at different $\omega_{0}$ and different scales. Through the experiments, we found that when the scale range was $\left[2^{5},2^{6}\right]$ and $\omega_{0}\;$= 8, we obtained the most suitable result feature.

**Figure 8:**
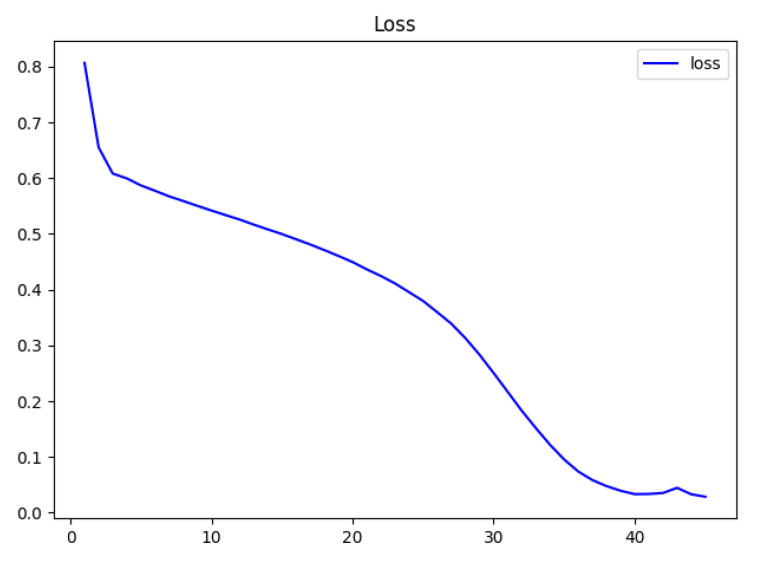
Loss function curve diagram. The *x*-axis is the epoch, and the *y*-axis is the loss. We save the loss value during the training process to draw the curve. According to the shape of the loss curve, we can analyze the quality of model training and adjust the parameters of the CNN. At the beginning of training, the loss function decreases rapidly and finally approaches 0 and converges.

**Figure 9:**
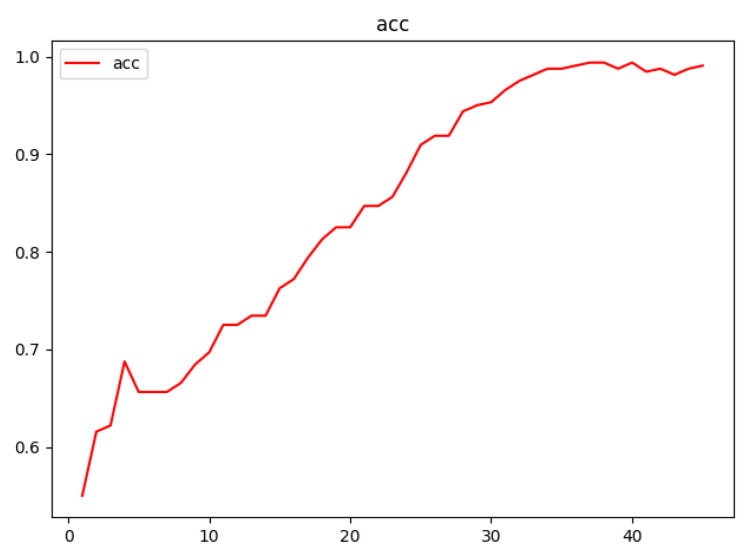
Accuracy curve diagram. The *x*-axis is the epoch, and the *y*-axis is the accuracy. The loss curve alone can provide little information, and the accuracy curve is generally used to determine whether it is over-fitting. At the beginning of training, the accuracy rises rapidly and finally approaches 1 and tends to converge.

The loss function is an operation function used to calculate the difference between the model’s predicted value, f(x), and the actual value, Y, during the model’s training phase. The smaller the loss function is, the better the robustness of the model is. The predicted value is output through forward propagation after each batch of training data has been fed into the model, and the loss function then works out the difference between the predicted value and the true value, which is the loss value. In order to achieve the goal of learning, the model updates each and every parameter by using back propagation after obtaining the loss value. This reduces the differences between the real value and the predicted value so that the predicted value it produces can be close to the real value. According to the loss function curve and acc accuracy curve in Figure 8 and Figure 9, the model developed performs well by using the dataset. The issue of over-fitting is resolved after parameter selection, and the loss curve is smooth and convergent. The acc curve showed a good learning state for the neural network because it increased quickly in the prior epochs. The loss curve and the accuracy curve tend to level off once the accuracy is close to 1, which shows that the model converges.

The precision, recall, and F1-score are typically used to evaluate a classification system model during testing. The percentage of relevant instances among all retrieved instances is what is referred to as precision. The percentage of retrieved instances among all pertinent instances is known as recall. The weighted average of precision and recall is known as the F1-score, and its calculation is as follows:

(11) $$F1=\left(\frac{2}{{\mathrm{recall}}^{-1}+{\mathrm{precision}}^{-1}}\right)=2\;\cdot \;\frac{\mathrm{precision}\;\cdot \;\mathrm{recall}}{\mathrm{precision}+\mathrm{recall}}$$

After the training was complete, we used the prepared test set to assess the model we trained. The test accuracy score for the entire model evaluation was 0.975. In the test, the model’s correct prediction ratio for the samples of chickens classified as abnormal was 0.96, and its correct prediction ratio for samples of chickens classified as normal was 0.99. The proportion that the model can correctly predict in all abnormal samples is 0.99, while it can correctly predict 0.96 in all normal samples. We also determined the model’s macro-avg and weighted-avg in order to assess it in additional ways. A thorough evaluation report of the model can be found in Table 3.

## 4. Discussion

Few studies have been carried out on the analysis and evaluation of livestock growth and production status by using hyperspectral technology in the early research of the livestock breeding industry. The majority of studies are based on the evaluation of livestock activity, behavior, and visual data. By securing the foot ring on each chicken, Bao and colleagues measured the three-dimensional displacement of the animal. They then designed and computed three-dimensional total variance to represent the animal’s level of activity. Finally, by taking a machine learning classification method, activity intensity is used to determine the condition of the chicken, enabling the determination of whether it is alive or dead [11]. In this technique, the chicken’s activity data are vulnerable to interference from the factory environment, human intervention, immune operation, and feeding restriction, which causes the system to misjudge and lowers judgment accuracy. A deep learning-based method for identifying sick chickens was proposed by Chen et al. They developed a model with a recall of 91.95%, an accuracy of 88.41%, and an F1-score of 89.93% by learning the fusion features of the posture features, body texture features, and head composite features of chicken images [9]. With this technique, sick chickens can be identified with high accuracy. Visual image recognition cannot reliably distinguish low-egg production laying hens from regular-laying hens due to the slight differences in their characteristics. Therefore, more research is needed to determine how to identify laying hens with low-egg production. The paper presents a general technique for identifying abnormal-laying hens using hyperspectral imaging technology and hyperspectral analysis. The comb’s hyperspectral data is acquired, and the VCA algorithm extracts the end members of the comb. FCWT is used to analyze the spectral curve, which is then transformed into a spectral feature image that is input into a straightforward convolutional neural network. The binary classification model developed during the experiment can successfully spot abnormal-laying hens with an accuracy of 0.975, an abnormal-laying hen identification precision of 0.96, a recall of 0.99, and an F1-score of 0.98. The FCWT wavelet transform’s high speed and high precision contribute to the proposed method’s accuracy and efficiency. This demonstrates the importance of the experiment’s analysis procedure for the use of hyperspectral imaging in other fields.

The analysis technique, which combines FCWT, deep learning, and hyperspectral technology, is also very important for determining the health status of chickens. The chicken’s comb is typically large, smooth, and bright red in color. A chicken with a disease has a comb that is abnormally colored, atrophic, or swollen and occasionally contains foreign objects. As a result, this method’s use in the analysis of chicken health and welfare can be further explored, and it also has some research and development value for other breeding industries, including pig, cattle, and sheep.

However, it should be noted that we used manual handheld devices to collect the hyperspectral data for the experiment detailed in this paper. This acquisition method’s flaw is that it requires a lengthy acquisition process, expensive labor, and a lot of acquisition time. It is possible to develop an integrated system that supports long-term continuous data collection while taking equipment costs into account in further research. It can reduce labor and time costs by automatically gathering data and identifying abnormal-laying hens on the farm throughout the entire period. It can also improve the efficiency of identifying abnormal-laying hens in laying hen breeding enterprises.

## 5. Conclusions

In this paper, we investigate a method for identifying abnormal-laying hens on farms using hyperspectral images, FCWT, and a convolutional neural network. We develop a deep learning binary classification model with a recognition accuracy of 0.975. Our results demonstrate that analyzing and extracting features from hyperspectral images can help create an accurate model for identifying laying hens with low-egg production in laying hen farms, which can improve animal welfare by identifying chicken health following adjustment. Furthermore, the method for analyzing hyperspectral image data based on FCWT proposed in this paper can inspire and inform further research on the use of hyperspectral technology in other fields.

Our data were gathered manually with the aid of portable devices during the experiment. In order to adapt to practical applications in the aquaculture industry, future research should focus on creating a large-scale integrated system that integrates automated data collection, analysis, and judgment functions.

## Figures and Tables

**Table 1:** Distribution table of comb dataset.

Total Cocks Spectrum Data	Normal-Egg Production	Low-Egg Production	Training Set	Validation Set
400	203	197	320	80

**Table 2:** Specification of CNN Net.

Configuration of the Best Performing Model	
Layers	Specification
Conv2D	Filters = 8, kernel_size = (3, 1), stride = (3, 1), activation = Relu
Maxpool-2D	pool_size = (2, 2), strides = (2, 2)
Conv2D	Filters = 32, kernel_size = (1, 1), stride = (1, 1), activation = Relu
Maxpool-2D	pool_size = (3, 3), strides = (2, 2)

**Table 3:** Model evaluation data sheet.

Parameters for Evaluation	Abnormal	Normal	Macro Avg	Weighted Avg
Precision	0.96	0.99	0.98	0.98
Recall	0.99	0.96	0.98	0.97
F1-score	0.98	0.97	0.97	0.97

## Data Availability

The chicken comb dataset and the FCWT code presented in this study are available in the Supplementary Material.

## Supplementary Materials

The following supporting information can be downloaded at: https://www.mdpi.com/article/10.3390/s23073645/s1, Figure S1: The environment of the cage in the chicken farm.
