# Supplementary material for: Recognition of Abnormal-Laying Hens Based on Fast Continuous Wavelet and Deep Learning Using Hyperspectral Images
Source: Sensors (Basel). 2023 Mar 31;23(7):3645. doi: 10.3390/s23073645 (PMC10098863; doi:10.3390/s23073645)
Supplement: Supplementary file 1 [file sensors-23-03645-s001.zip › sensors-2292232-supplementary.pdf]

supplementary material

Recognition of Abnormal-Laying Hens Based on Fast Continuous Wavelet and Deep Learning Using Hyperspectral Images

Xing Qin [1], Chenxiao Lai [1], Zejun Pan [1], Mingzhong Pan [2], Yun Xiang [3] and Yikun Wang [2,*]

[1] Zhejiang Key Laboratory of Large-Scale Integrated Circuit Design, Hangzhou Dianzi University
[2] School of Physics and Photoelectric Engineering, Key Laboratory of Gravitational Wave Precision Measurement of Zhejiang Province, Taiji Laboratory for Gravitational Wave Universe, Hangzhou Institute for Advanced Study, University of Chinese Academy of Sciences, Hangzhou 310024, China
[3] Agriculture science research institute Jinhua city Zhejiang province
* Correspondence: wangyikun@ucas.ac.cn;

Information about animal

Large-scale chicken farming is conducted in our experimental ranch, which is situated in Zhangjia Village, Yongchang Street, Lanxi, Jinhua City, Zhejiang Province, China. 80 000 laying hens were raised there.The feed ratio is Yellow corn seed (63%), bean meal (24%), mountain flour (6%), soybean oil (0.5%), pepper meal (2.5%) and premix (4%). In order to achieve automatic environmental control, automatic feeding, drip-tube drinking water, automatic transmission of chicken excrement, etc., it employs the cage feeding method. In essence, this achieves automatic breeding. The environment of the cage in the chicken farm is shown in Figure S1. The biggest task each day is to watch the fence, though. It has been determined that sick hens with a low egg production rate should be raised separately or destroyed. We have also done research, and there are just too many of these kinds of poultry farms in China. The farm managers think that one of the biggest problems is figuring out how to discover sick birds or chicks with low egg production.

The goal of this paper's research is to develop a clever system that can locate sick chickens with low egg production rates automatically, alert the breeder via the system, and direct the breeder to the appropriate treatment. On the one hand, it lessens the breeders' work requirements. On the other side, it collects information about deceased hens or hens with a decreased egg production rate, which helps businesses make judgments.

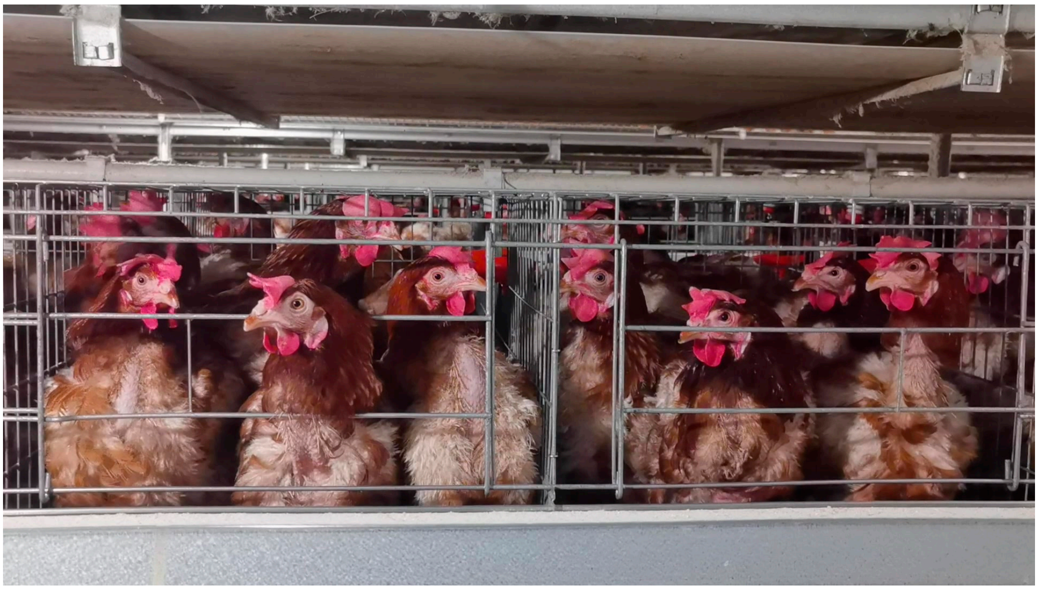

Figure S1. The environment of the cage in the chicken farm.

Comb Spectral Dataset

https://github.com/LaiChauncy/Comb-Dataset.git

The Fast Continuous Wavelet Transformation

https://github.com/LaiChauncy/mat_fcwt.git
